# Dynamical analysis of the global business-cycle synchronization

**DOI:** 10.1371/journal.pone.0191491

**Published:** 2018-02-06

**Authors:** António M. Lopes, J. A. Tenreiro Machado, John S. Huffstot, Maria Eugénia Mata

**Affiliations:** 1 UISPA–LAETA/INEGI, Faculty of Engineering, University of Porto, Porto, Portugal; 2 Institute of Engineering, Polytechnic of Porto, Dept. of Electrical Engineering, Porto, Portugal; 3 Nova School of Business and Economics, Faculdade de Economia, Universidade Nova de Lisboa, Lisbon, Portugal; China University of Mining and Technology, CHINA

## Abstract

This paper reports the dynamical analysis of the business cycles of 12 (developed and developing) countries over the last 56 years by applying computational techniques used for tackling complex systems. They reveal long-term convergence and country-level interconnections because of close contagion effects caused by bilateral networking exposure. Interconnectivity determines the magnitude of cross-border impacts. Local features and shock propagation complexity also may be true engines for local configuration of cycles. The algorithmic modeling proves to represent a solid approach to study the complex dynamics involved in the world economies.

## 1 Introduction

Classic models describe natural and artificial systems using the formalism of mathematics and physics. In the last decades emerged the alternative of computational modeling, particularly in cases where the complexity, or the type, of the phenomena involved makes it difficult to adopt standard methodologies. We find examples of such modeling exercises in several disciplines such as engineering, biology, geophysics, finance and economy, just to name a few [1, 2]. Models provide a guideline for our understanding of the phenomena involved, highlighting the system main characteristics, and the most important variables and parameters. Classic methodologies are supported by differential calculus, leading to the concept of dynamics, and contributing to predictions of the evolution of the state variables. Mathematical models are being applied to economics, but the complexity of this area, involving a plethora of factors such as political, social, environmental, and others, leads to descriptions not matching real-world observations [3–7]. The computational perspective supports a powerful alternative for describing complex phenomena from the point of view of time series analysis. This option is based on real-world data and adopts assertive algorithms supported by solid mathematical tools [8–12]. This paper follows this second strategy for studying the world economy dynamics during the last half century. For that purpose a sample of 12 countries is considered and the study analyzes possible effects of globalization based on the evolution of their time series [13–21]. Due to the lack of data for all countries, it is considered that taking a sample of countries is a more reliable strategy than estimating values for long periods of time of other countries. Nonetheless, analyzing a number of countries during a long time period poses challenges that are tackled by means of advanced algorithmic tools.

Baxter and Kouparitsas [22] begin their paper saying “When America sneezes, Europe catches a cold”. It may be, however, that not all colds are caught from the same sneeze, and in fact, we present evidence below suggesting that there are many sneezes. This is not to deny that co-movement with the business cycles of the United States exists, but to report empirical support for the fact that local economies sometimes “go their own way”. As world economic hegemony belongs to the United States of America, this paper reflects on the synchronization of economic fluctuations in the mirror of the United States’ behavior, by examining patterns of biased positions among them. Globalization has boosted bilateral trade and contagion effects, as seen in the Gross Domestic Product (GDP) of countries, and commercial flows appear to confirm the hypothesis of ever greater synchronization of economic performance. Proximity of neighboring countries and cross-border openness induce trade contagion effects. International shocks may also be safely considered as disturbances or at least as sources of some contagion. The greater the openness, the greater too will be the contagion effects from global linkages. Ties with other countries in open economic integration (such as the European Community, the MERCOSUL, or the Andean Community) may also be pointed to as factors of contagion and co-movement, in times of prosperity and in recessions. Intensified international relationships among the partners of international organizations may increase dependence on foreign demand for exports, which is driven by the prosperity of those foreign members. Financial openness is another element that brings simultaneity and cycle synchronization [23].

Interconnectedness matters, as the theory of optimum currency areas demonstrates [24]. Nevertheless, co-operation can also introduce coordinated policies to minimize negative impacts among country partners, even without political union, because “In the real world, of course, currencies are mainly an expression of national sovereignty” [25]. A common monetary policy and financial integration may even be sources of international shocks, according to Rose and Engel [24].

Countries having the most similar sector structure in their economies will also have the most similar business cycle behavior [26]. Kydland and Prescott [27] introduce the real business-cycle theory, and Backus et al. [28] extend this theory across countries for output (and consumption). The Heckscher-Ohlin theory highlights the importance of endogenously-produced factor endowments (such as human capital and using technical education, for example), in any national economy. However, if there is only one sector imbalance resulting from an external shock, the other sectors in the national economy may respond by striving for greater specialization to take advantage of the situation. Their increasing returns to scale may offset the negative effects of the external shock for the business cycle, as Helpman and Krugman [29] demonstrate. The ambiguity inherent in this situation demands careful empirical checking. Artis et al. [30] conclude that “The large body of research that explored (…) changes on business cycle behavior has produced mixed results”.

Kose et al. [31–33] remind us that the performance observed for many developing economies does not confirm the business cycle synchronization hypothesis for GDP in the context of increasing international trade and financial market integration. On the contrary, for industrialized Europe, Artis et al. [34], Artis and Zhang [35] and Artis [36] confirm the existence of a GDP “European business cycle” since the early 1980s. A theory on GDP business cycle clubs superseding European (or regional business cycles) is also advanced in Artis et al. [37]. Correlations of decreasing output in recent decades among the major industrial countries showing remarkable de-synchronization in the late 1980s and early 1990s are available in Helbling and Bayoumi [38] and Doyle and Faust [39].

Unemployment is the most visible and well-known feature of business-cycle busts. Demographic variables have been less considered in the literature, but they deserve to be included, because social exclusion increases death rates in business-cycle crises, and poor pre-natal nutrition during periods of downturn can thereby cancel out some collective achievements regarding welfare and life expectancy at birth. Life expectancy is a reasonable reflection of a country’s overall welfare, which itself reflects the soundness of the local economic environment. Demographic variables also emerge as a good proxy for business cycles, because migration is the consequence of attraction or repulsion of the labor force in job markets (i.e., the local economic environment), especially in a context of free labor movements and low information and transportation costs. Life expectancy and birth rates are nodes of stylized vulnerabilities, because of the impact of busts on demography and their dampening effect on migration.

The literature above disregards these issues. Because national economies vary tremendously in size, the current research also includes GDP *per capita*, thereby avoiding the risk of scale bias in the conclusions.

A wide range of developing economies does not confirm the business cycle synchronization hypothesis for GDP in the context of increasing international trade and financial market integration, according to Kose et al. [31, 32]. On the contrary, for industrialized Europe, Artis and Zhang [34–36] confirm the existence of a GDP “European business cycle” since the early 1980s. A theory on GDP business cycle clubs superseding European (or regional business cycles) is also available in [37, 40]. Nonetheless, it is also said that business cycle co-movements increased during USA recessions across industrialized countries [41] and across industrialized and developing countries [42, 43]. Antonakakis [44] concludes that “the 2007-2009 recession, compared to any of the 30 recession episodes which occurred since the 1870s in the USA, increased business cycle synchronization across the G7 countries to unprecedented levels”. Because countries have different national-economy dimensions, the current research uses GDP *per capita* growth rates, which is appropriate for international comparisons to avoid the problem that scale effects may bias the conclusions on the identification of business-cycles. However, the analysis of one single variable, GDP, is too narrow a perspective to define business-cycles of national economies [45]. We include the role of openness and trade to proxy contagion effects, and exports *per capita* are used in order to proxy the domestic surpluses related with foreign demand of national products that frame openness.

The intensification of globalization after the Second World War justifies our observation of the period from then until the present. Seeking to cover the behavior of the great partners of the developed and developing world, we examine the major global economies having continuous time series in the World Bank database for a considerable number of years. Twelve countries satisfy these conditions (Australia, Brazil, Canada, China, France, India, Japan, Mexico, South Africa, Switzerland, the United Kingdom, and the United States) for the 56 years from 1960 to 2015. The importance of detecting patterns of economic performance synchronization among these partners in a comparative perspective with the USA is undeniable. Greater synchronization of the countries of the Organization for Economic Co-operation and Development (OECD) with the USA, or among those countries themselves, may explain degrees of market efficiency in response to real shocks, or may require different coordination levels of Keynesian macroeconomic policies during depressions to avoid selfish national policies or regional xenophobia from perpetuating crises, as occurred with the Great Depression of 1929-1933.

These considerations follow the heuristic reasoning inspired by available economic thinking. Nevertheless, the question remains about having a quantitative methodology that supports such a perspective based on factual evidence. Historical data reflect many economic and social features in the space of national economies. Consequently, they can be interpreted as manifestations, or “outputs”, of complex dynamic systems to be interpreted according to macroeconomics.

This paper is innovative in two ways: the choice of variables to examine, comprising GDP *per capita*, the weight of Exports in GDP as a percentage, and life expectancy at birth; and the computational means by which social features are combined with economic indicators and treated as performance outputs of a dynamic system, and then compared to the corresponding outputs of the USA.

The paper is organized in the following way. Section 2 introduces the main aspects of the historical and economic world development. Section 3 presents sources of data and country details. Section 4 describes the mathematical methodology for business cycles based on a multiple-variable approach. Section 5 analyzes and discusses the results. Finally, Section 6 outlines the main conclusions in checking the historical narrative of the main business cycles.

## 2 Timeline of economic evolution

**Fig 1:**
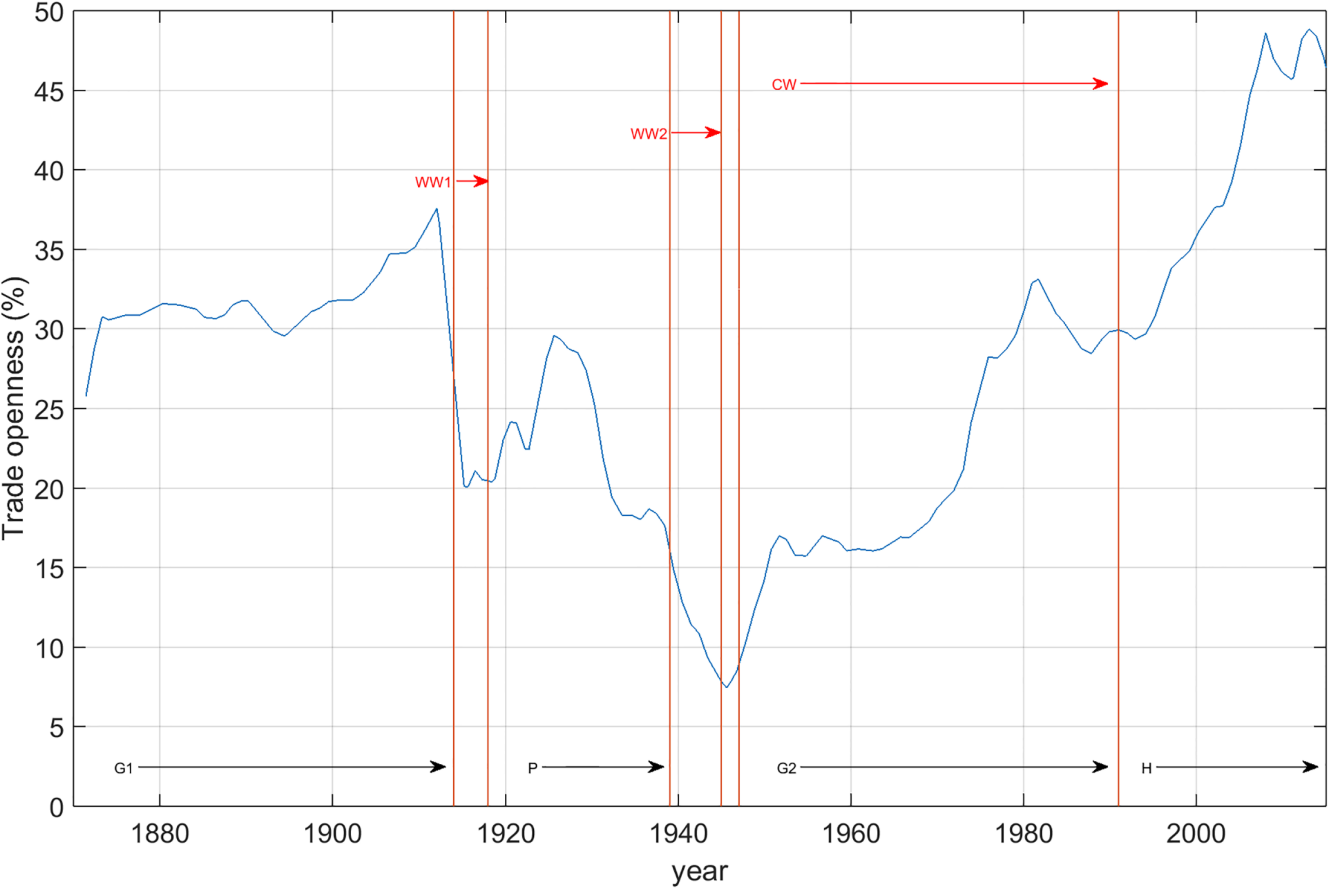
Timeline of globalization (1880-2015), proxied by trade openness, calculated as (exports + imports) as percentage of GDP [46]: WW1—First World War; WW2—Second World War; CW—Cold War; G1—First phase of globalization; P—Protectionism; G2—Second phase of globalization; H—Hyper-globalization.

**Fig 2:**
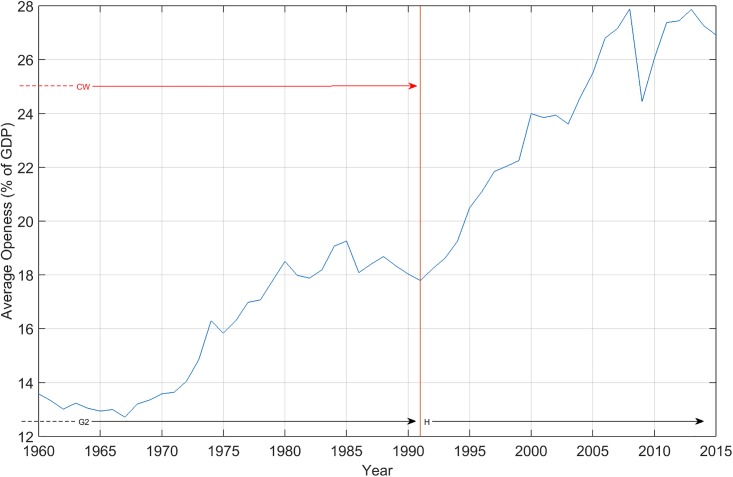
Timeline of globalization (1960-2015), proxied by the average openness of the 12 countries sampled in this paper.

Globalization in a long-run perspective [46] may consider openness of national economies to international markets, as in Fig 1. The peaceful period before the First World War (WW1) was tremendously successful under the gold-standard monetary regime, with free movements of commodities, labor, and capital, in benefiting from trans-continental cross-border railroads, steam-driven shipping, and telegraph (G1). On the contrary, the bellicose 1914-1945 period saw a clear de-globalization character under flexible exchange rates, protectionism (P), and closed-door policies for migration. Again, peaceful Cold War (CW) times after the Second World War (WW2), under the Bretton-Woods fixed-exchange rates, air transportation, and container-shipping amounted to a new globalization wave (G2), especially after the difficulties of the crisis of the late 1980s. It is well known that the USA presented a smooth evolution before 1980. The performance of the USA and the stability of its global performance and social welfare in the post-war period (from 1948 to 1973) with full employment were remarkable [47]. Business-cycle fluctuations were quite flat and overcome promptly, even when fearing consequences from the Korean war in the early 1950s, the Suez crisis in 1956, and the dollar inconvertibility in 1971 [47]. Economists even forgot business-cycle theories, according to Krugman [48]. For the “Great Moderation” in the USA economy, see Spatafora and Sommer [49]. Capitalism expansion to regions of the old Soviet Union and China in the 1990s, and technological progress, have contributed to a hyper-globalization phase (H). The 2008 crisis may be the turning point for a new de-globalization phase. The effects of the Iraq and Afghanistan wars on the American business-cycle behavior, the 1998 panic crisis, George Soros’ speculations, the Fannie Mae and Freddie Mac boom events of 2004-2006, and the 2008 Lehman Brothers collapse, are all American features establishing a pattern, and are the mirror for comparing other partners’ business-cycle behaviors. For the 12 countries sampled in this paper, Fig 2 confirms these conclusions. The data available (until 2015) may hint at the evolution to follow.

Indicator Exports of goods and services (% of GDP) (NE.EXP.GNFS.ZS) corresponds to the proxies openness in Fig 2. Exports of goods and services represent the value of all goods and other market services provided to the rest of the world. They include the value of merchandise, freight, insurance, transport, travel, royalties, license fees, and other services, such as communication, construction, financial information, and business, personal, and government services. They exclude compensation of employees’ and investment income (formerly called factor services) and transfer payments. Source World Bank national accounts data, and OECD National Accounts data files.

## 3 Database sources

The principal data in our study come from real-world time series. The final objects to be analyzed have the following characteristics: 3 economy indices, 12 countries, and 56 years.

Data were collected from the World Bank national development indicators, http://data.worldbank.org/data-catalog/world-development-indicators. The indices used cover exactly the period from 1960 to 2015, addressing the variables, *x*_*k*,*i*_(*t*), *k* = 1, ⋯, 3, *i* = 1, ⋯, 12, *t* = 1960, ⋯, 2015. At the time of writing, data for year 2016 were not yet available. The details of the indices are as follow:

- *x*_1,*i*_(*t*) = {GDP *per capita* (constant 2010 USD)}—The GDP *per capita* comes from NY.GDP.PCAP.KD. It is the GNI *per capita* (constant 2010 USD). GNI *per capita* is gross national income divided by midyear population. GNI (formerly GNP) is the sum of value added by all resident producers plus any product taxes (less subsidies) not included in the valuation of output plus net receipts of primary income (compensation of employees’ and property income) from abroad. Data are in constant 2010 USD.
- *x*_2,*i*_(*t*) = {Exports of goods and services (% of GDP)}—The annual exports of goods and services (% of GDP) comes from NE.EXP.GNFS.ZS. Exports of goods and services represent the value of all goods and other market services provided to the rest of the world. They include the value of merchandise, freight, insurance, transport, travel, royalties, license fees, and other services, such as communication, construction, financial information and business, personal, and government services. They exclude compensation of employees’ and investment income (formerly called factor services) and transfer payments. It is a weighted average. Data are expressed as a percentage of GDP.
- *x*_3,*i*_(*t*) = {Life expectancy at birth, total (years)}—The life expectancy at birth comes from SP.DYN.LE00.IN, and indicates the number of years a newborn infant would live if prevailing patterns of mortality at the time of its birth were to remain the same throughout its life. Derived from male and female life expectancy at birth from sources such as: (1) United Nations Population Division. World Population Prospects, (2) United Nations Statistical Division. Population and Vital Statistics Report (various years), (3) Census reports and other statistical publications from national statistical offices, (4) Eurostat: Demographic Statistics, (5) Secretariat of the Pacific Community: Statistics and Demography Program, and (6) USA Census Bureau: International Database. The data are in years.

The 12 countries considered in this study are {Australia, Brazil, Canada, Switzerland, China, France, United Kingdom, India, Japan, Mexico, South Africa, United States}, to be denoted in the hereinafter by the acronyms {AUS, BRA, CAN, CHE, CHN, FRA, GBR, IND, JPN, MEX, ZAF, USA}. This strategy defines a space-time of analysis, involving 3 (social-economic indices)×12 (countries)×56 (years). We shall follow several modeling perspectives in order to construct a robust mathematical description.

## 4 Methodology

Two strategies are considered for system analysis in processing the data evolution over time and are addressed in the next two sub-sections: a mathematical and computational description by means of (i) state space (SS) representation, and (ii) *relativistic* perspective, based on distances between countries and a reference (the USA).

### 4.1 The state space approach

The first approach, which is the SS representation, is a well-known method in the study of dynamic systems and consists of having time as a parametric variable, and the other variables as the main ones [10, 50]. With this tool we interpret the economic variables as outputs of a complex system and the state variables as representative of the major phenomena required to develop a *model*, in which the length, depth, and shape of business-cycles may be different. For a methodology to disentangle these aspects using duration, amplitude, and excess as measures, see [51].

For constructing the *n*-dim SS portrait we use the time series, *x*_*k*,*i*_(*t*), *k* = 1, ⋯, 3, *i* = 1, ⋯, 12, and its 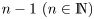 time derivatives, often called phase variables. The adoption of a *n*-dim representation is a compromise between assertiveness and feasibility, and in practice we often choose *n* ≤ 3 since visualization techniques are limited to 3-dim charts.

The numerical calculation of the time derivatives needs a careful design of the algorithm since noise may occur, producing artifacts in the resulting signal. We adopt the algorithm proposed in [52]:

$$\begin{array}{c} {\dot{x}}_{k,i}(t)=\frac{1}{8h}\{2[x_{k,i}(t+h)-x_{k,i}(t-h)]+x_{k,i}(t+2h)-x_{k,i}(t-2h)\}, \end{array}$$ (1a)

$$\begin{array}{c} {\ddot{x}}_{k,i}(t)=\frac{1}{4h^{2}}\{[x_{k,i}(t+2h)+x_{k,i}(t-2h)]-2x_{k,i}(t)\}, \end{array}$$ (1b)

where *h* denotes the sampling period (in our case *h* = 1 year).

Expression (1) requires two additional points at left and right that were obtained by extrapolating *x*_*k*,*i*_(*t*). Several numerical experiments demonstrated that these formulae produce good results for the time series under analysis.

**Fig 3:**
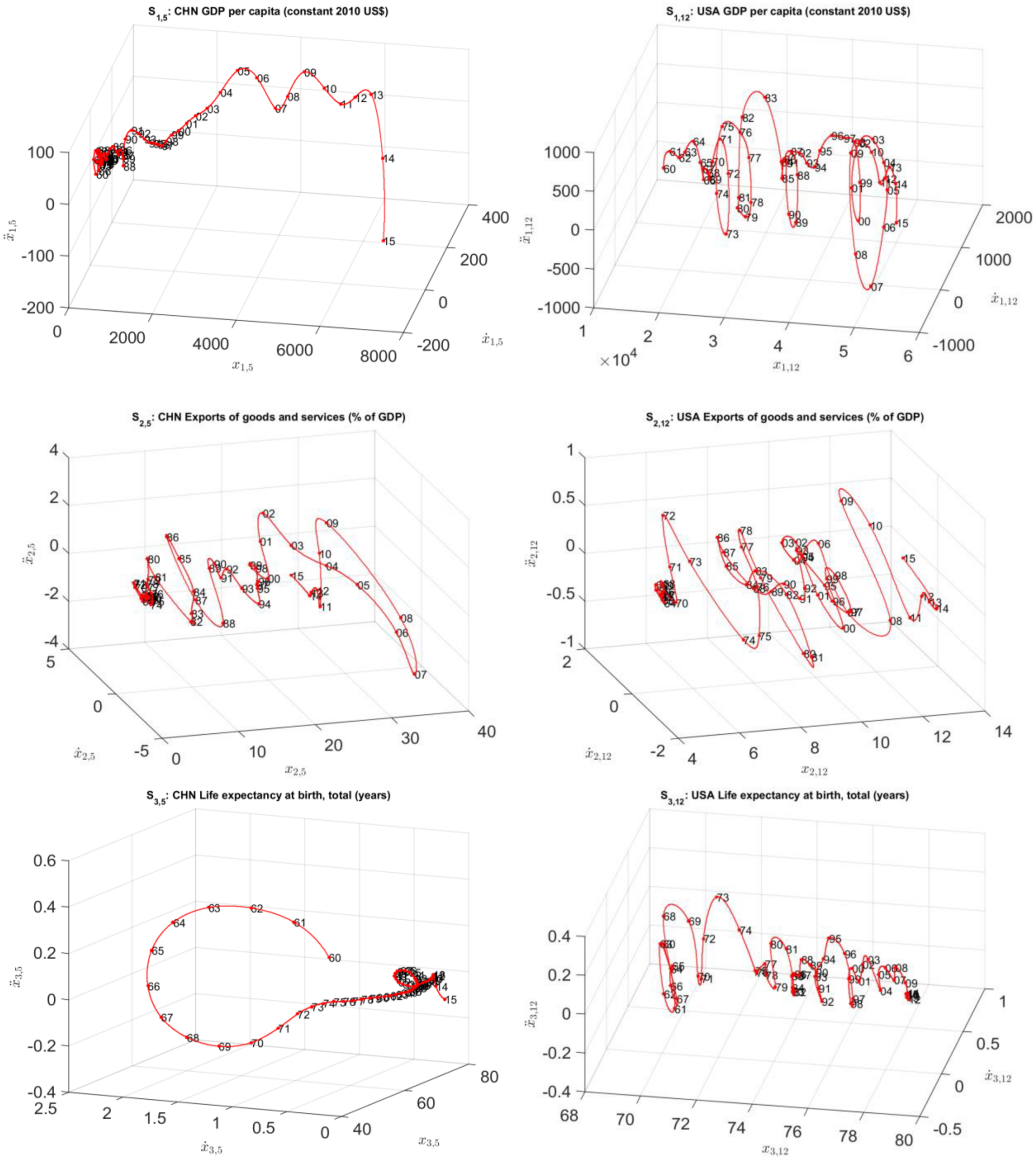
The 3-dim SS trajectories, *S*_*k*,*i*_, *k* = 1, ⋯, 3, *i* = {5, 12}, for China and USA, *t* ∈ [1960, 2015].

Fig 3 depicts the 3-dim SS trajectories (to be denoted in the hereinafter by *S*_*k*,*i*_) for the time series *x*_*k*,*i*_(*t*), *k* = 1, ⋯, 3, and *i* = {5, 12}, corresponding to China and USA, respectively. The “main loops” describe historical periods of fluctuations. For example, for the SS of the USA GDP *per capita*, *S*_1,12_, we observe loops close to years {1980, 1986, 1990, 2000, 2007}, which correspond to the 1980 recession, the 1986 tax cut, the 1990-1991 recession, the 2001 9/11 terrorist attacks, and the 2007-2008 banks and financial crisis, respectively.

The use of 3-dim SS charts highlights the role of acceleration and emphasizes the variations in the velocity. For example, in the USA (for index *k* = 1), and China (for index *k* = 3), we verify the existence of dynamical loops in recent years that are incompletly represented in 2-dim charts. However, a precise and assertive visualization of points and coordinates reveals difficulties that are straightforward to overcome with 2-dim charts. Therefore, we decided to explore two strategies: the comparison of the complete charts by means of HC and the analysis of SS using 2-dim projections.

#### 4.1.1 Hierarchical clustering analysis of the SS trajectories

For each variable we compare the 3-dim SS trajectories of all countries, and we adopt hierarchical clustering (HC) for illustrating the results. HC is a technique for analyzing and visualizing relationships embedded in data. The algorithm generates groups of objects that are similar to each other in some sense, and generates a graphical representation of the objects in the form of a dendrogram or cluster tree [53].

The HC processes the matrix **M** = [*H*(*S*_*k*,*i*_, *S*_*k*,*j*_)], where *H*(*S*_*k*,*i*_, *S*_*k*,*j*_) is the Hausdorff distance [54] between the SS trajectories *S*_*k*,*i*_ and *S*_*k*,*j*_, *k* = 1, ⋯, 3, *i*, *j* = 1, ⋯, 12:

$$\begin{array}{c} H\left(S_{k,i},S_{k,j}\right)=\max\left\{h\left(S_{k,i},S_{k,j}\right),h\left(S_{k,j},S_{k,i}\right)\right\}, \end{array}$$ (2)

where *h*(*S*_*k*,*i*_, *S*_*k*,*j*_), or *h*(*S*_*k*,*j*_, *S*_*k*,*i*_), represents the directional Hausdorff distance between *S*_*k*,*i*_ and *S*_*k*,*j*_, or *S*_*k*,*j*_ and *S*_*k*,*i*_, given by:

$$\begin{array}{c} h\left(S_{k,i},S_{k,j}\right)=\max_{p\in S_{k,i}}\{\min_{q\in S_{k,j}}∥p-q∥\}, \end{array}$$ (3)

where *p* and *q* are points of *S*_*k*,*i*_ and *S*_*k*,*j*_, respectively.

Intuitively, *h*(*S*_*k*,*i*_, *S*_*k*,*j*_) finds the point *p* in the set *S*_*k*,*i*_ that is farthest from any point in set *S*_*k*,*j*_, and measures the distance from *p* to its nearest neighbor in *S*_*k*,*j*_.

**Fig 4:**
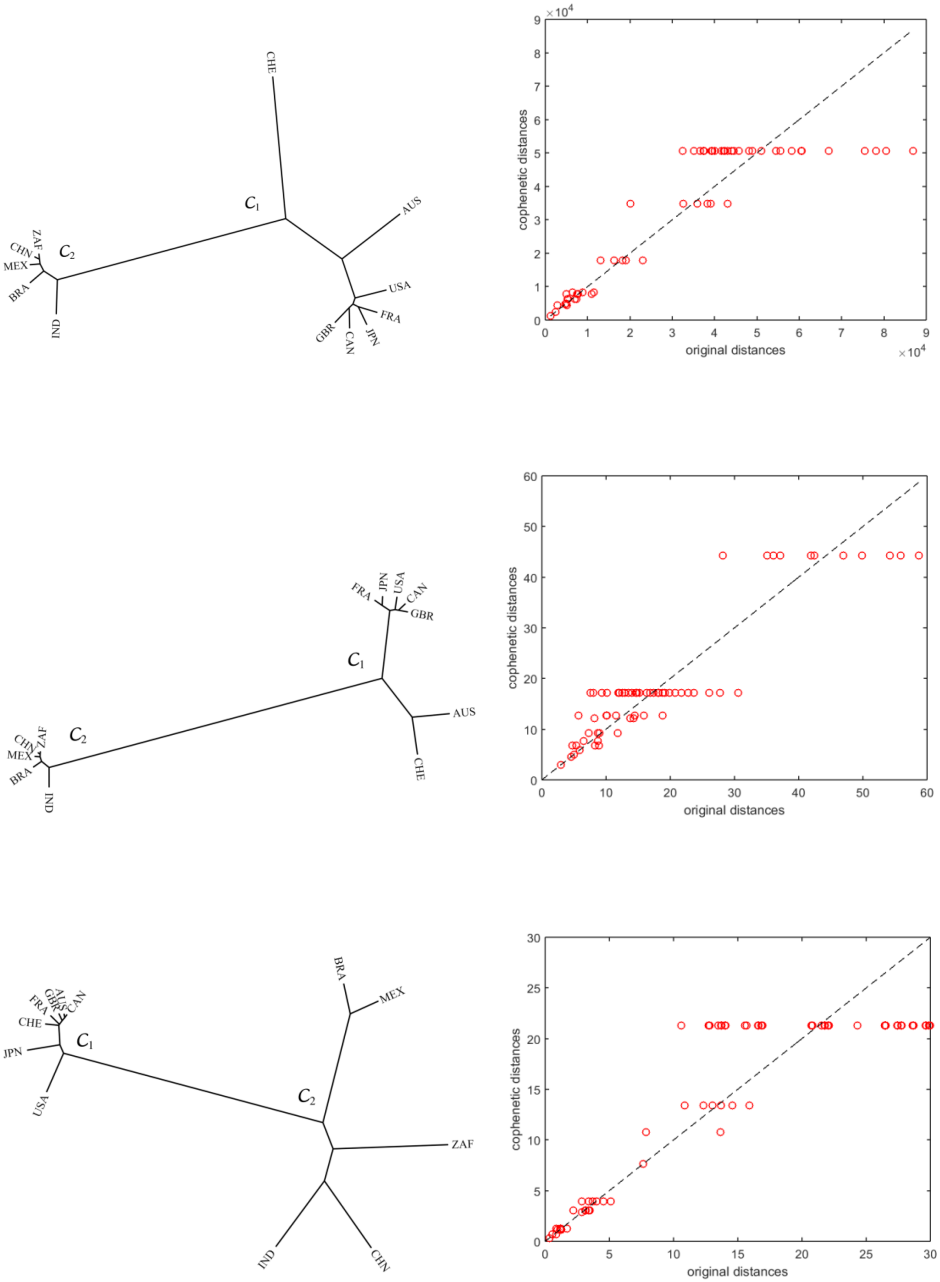
The hierarchical trees and the corresponding Shepard plots, generated by the HC with matrix M, for the variables GDP *per capita*, annual exports, and life expectancy, *t* ∈ [1960, 2015].

Fig 4 depicts the cluster trees generated by applying the successive (agglomerative) and average-linkage methods [53, 55, 56] for the variables GDP *per capita*, annual exports, and life expectancy, in the period 1960-2015. All trees reveal identical clusters, namely $C_{1}$ = {AUS, CAN, CHE, FRA, GBR, JPN, USA} and $C_{2}$ = {BRA, CHN, IND, MEX, ZAF}, composed of developed and developing countries, respectively, meaning that there the variables are correlated.

To assess the reliability of the clustering we use Shepard plots that compare the original and the cophenetic distances [57]. The better a cluster tree reflects matrix **M**, the closer to the 45 degree line the scatter points will lie. Therefore, from the charts of Fig 4 we conclude that the cluster trees accurately represent the original data in **M**.

#### 4.1.2 Analysis of the 2-dim SS trajectories

**Fig 5:**
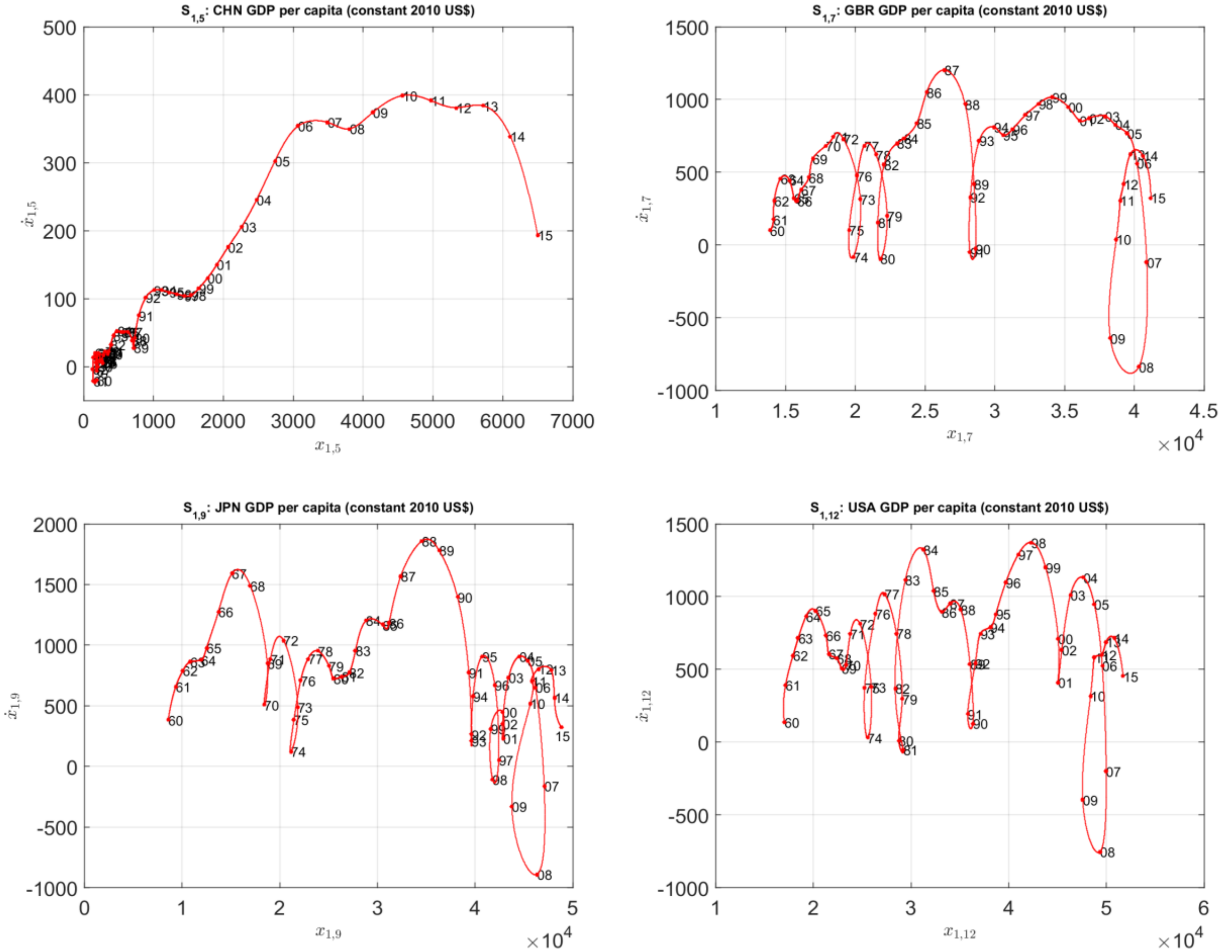
The 2-dim SS trajectories for the GDP *per capita* the countries {CHN, GBR, JPN, USA}, *t* ∈ [1960, 2015].

The 3-dim SS charts reveal the dynamical evolution of the variables. However, some details are better understood when looking to the 2-dim projections. For example, Fig 5 reproduces the 2-dim SS trajectories for the GDP *per capita* of the countries {CHN, GBR, JPN, USA}. The four countries experienced recession in 2008-2009, which was more severe in USA and GBR (Fig 5).

According to data, we observe that the 2008-2013 period brought a tremendous shock to all USA partners and great GDP losses. After the financial crisis, the effects on globalization go on: “the volumes of the bilateral linkages contracted sharply, due to the reduction in counterparty exposures and the de-leveraging processes that ensued as endogenous and procyclical responses to the financial crisis” [58]. Closing-door policies generally followed the previous increase in volume of the bilateral linkages in the macro network of the hyper-globalization that followed the late 1980s. The financial shock propagation and its aftermath have been accompanied by military conflicts that sum-up without resolution. Conflicts in Iraqi, Afghanistan, Syria, Turkey, Somalia, Nigeria, Darfur, Libya, Yemen, and Sudan, have made large migrant waves of refugees, not to mention the permanent Israeli-Palestinian problems, guerrilla warfare and terrorism [59].

The reinvigoration of global growth intended by global institutions, such as the IMF and the World Bank, clash with politicians’ reluctance to face the increasing public opinion wave that is supportive of autarchy and self-sufficiency. Emerging market economies can maintain their exports in demand, but the multinationals from the most developed countries are losing the opportunities of the hyper-globalization cheap labor, low taxing, de-regulation, and free markets.

### 4.2 The *relativistic* perspective

The second approach for the computational and mathematical description of the economy dynamics is the distance-based methodology, which consists of adopting a *relativistic* perspective instead of the standard absolute coordinate system description [60, 61]. In other words, when using distances between two objects, we are interpreting values on the basis of a comparison between items. For reference the USA time series are adopted, given the importance of its economy and influence upon the rest of the world.

#### 4.2.1 Standard determinants of business cycle

Standard determinants of business cycle co-movement include international trade, *T*_*i*,*j*_(*t*), specialization, *S*_*i*,*j*_(*t*), and financial factors, *F*_*i*,*j*_(*t*). Recently, Ductor and Leiva-Leon [62] showed that government expenditure, *Z*_*i*,*j*_(*t*), (a proxy of fiscal policy) and factor endowments, *W*_*i*,*j*_(*t*), such as human capital, are also important for explaining changes in business cycle interdependence. These five determinants, for countries *i* and *j*, and time instant *t*, are defined as [62]:

- $T_{i,j}\left(t\right)=\frac{E_{i,j}\left(t\right)+I_{i,j}\left(t\right)}{G_{i}\left(t\right)+G_{j}\left(t\right)}$, where *E*_*i*,*j*_(*t*) and *I*_*i*,*j*_(*t*) denote the total Exports and Imports from (to) country *i* to (from) country *j*, and *G*_*i*_(*t*) is the nominal GDP of country *i*, in year *t*. This determinant is computed using data from the IMF direction of trade statistics (https://data.world/imf/direction-of-trade-statistics-dots), for *E*_*i*,*j*_(*t*) and *I*_*i*,*j*_(*t*), and the World Bank national development indicators, for *G*_*i*_(*t*) (which corresponds to the GDP in constant 2010 USD).
- $S_{i,j}\left(t\right)=\sum_{p=1}^{P}\left|S_{i}^{p}\left(t\right)-S_{j}^{p}\left(t\right)\right|$, where $S_{i}^{p}\left(t\right)$ represents the GDP share of sector *p* = 1, 2, 3, for country *i* and year *t*. We use Agriculture, Industry, and Services value added, in percentage of the GDP, obtained from the World Bank national development indicators.
- $F_{i,j}\left(t\right)=\frac{A_{i}\left(t\right)+L_{i}\left(t\right)}{G_{i}\left(t\right)}+\frac{A_{j}\left(t\right)+L_{j}\left(t\right)}{G_{j}\left(t\right)}$. For the variables *A*_*i*_(*t*) and *L*_*i*_(*t*) we adopt the Bank capital to assets ratio and the Bank liquid reserves to bank assets ratio, respectively, as given by the World Bank national development indicators.
- $Z_{i,j}\left(t\right)=\sum_{p=1}^{P}\left|Z_{i}^{p}\left(t\right)-Z_{j}^{p}\left(t\right)\right|$, where for $Z_{i}^{p}\left(t\right)$, *p* = 1, 2, we adopt the Agricultural machinery tractors per 100 km^2^ of arable land, and the Agricultural land (in percentage of land area). The data come from the World Bank national development indicators.
- $W_{i,j}\left(t\right)=\sum_{p=1}^{P}\left|W_{i}^{p}\left(t\right)-W_{j}^{p}\left(t\right)\right|$, where for *W*_*i*_(*t*), *p* = 1, 2, we adopt the Goods and services expense (in percentage of expense), and Interest payments (in percentage of revenue). The data come from the World Bank national development indicators.

We calculate the determinants for each country, *i* = 1, ⋯, 11, relative to the USA, *j* = 12, and *t* ∈ [1960, 2015]. For each determinant, we approximate the 11 country trajectories by means of exponential functions:

$$\begin{array}{c} T_{i,j}\left(t\right)=a\exp(-bt),\;a,b\in R^{+}, \end{array}$$ (4)

with parameters *a* and *b* estimated numerically.

**Fig 6:**
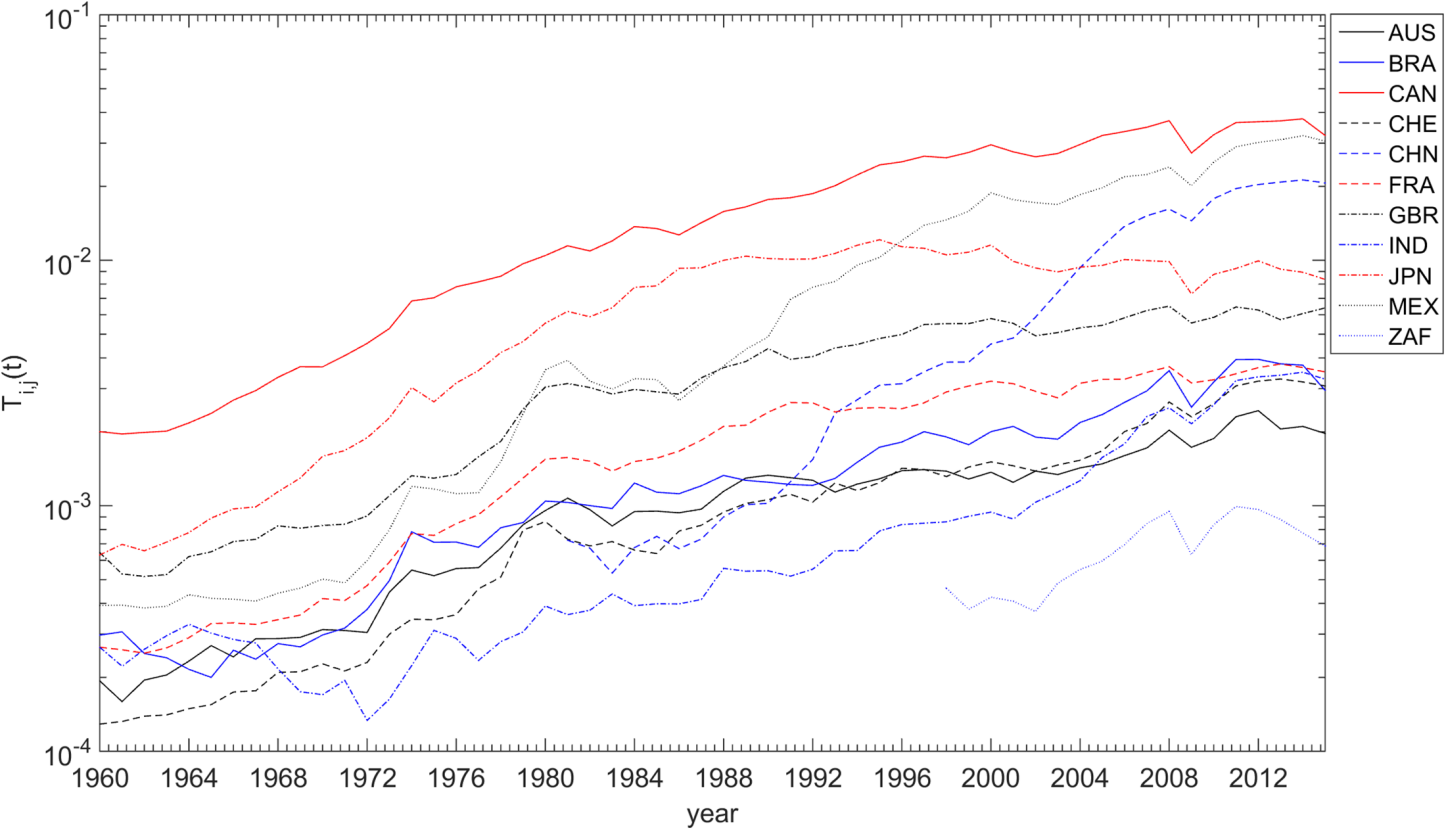
The international trade, *T*_*i*,*j*_(*t*), versus time, *t* ∈ [1960, 2015], for each of the 11 countries analyzed, relative to the USA.

For example, Fig 6 depicts *T*_*i*,*j*_(*t*). Some gaps in the curves are due to missing data.

**Fig 7:**
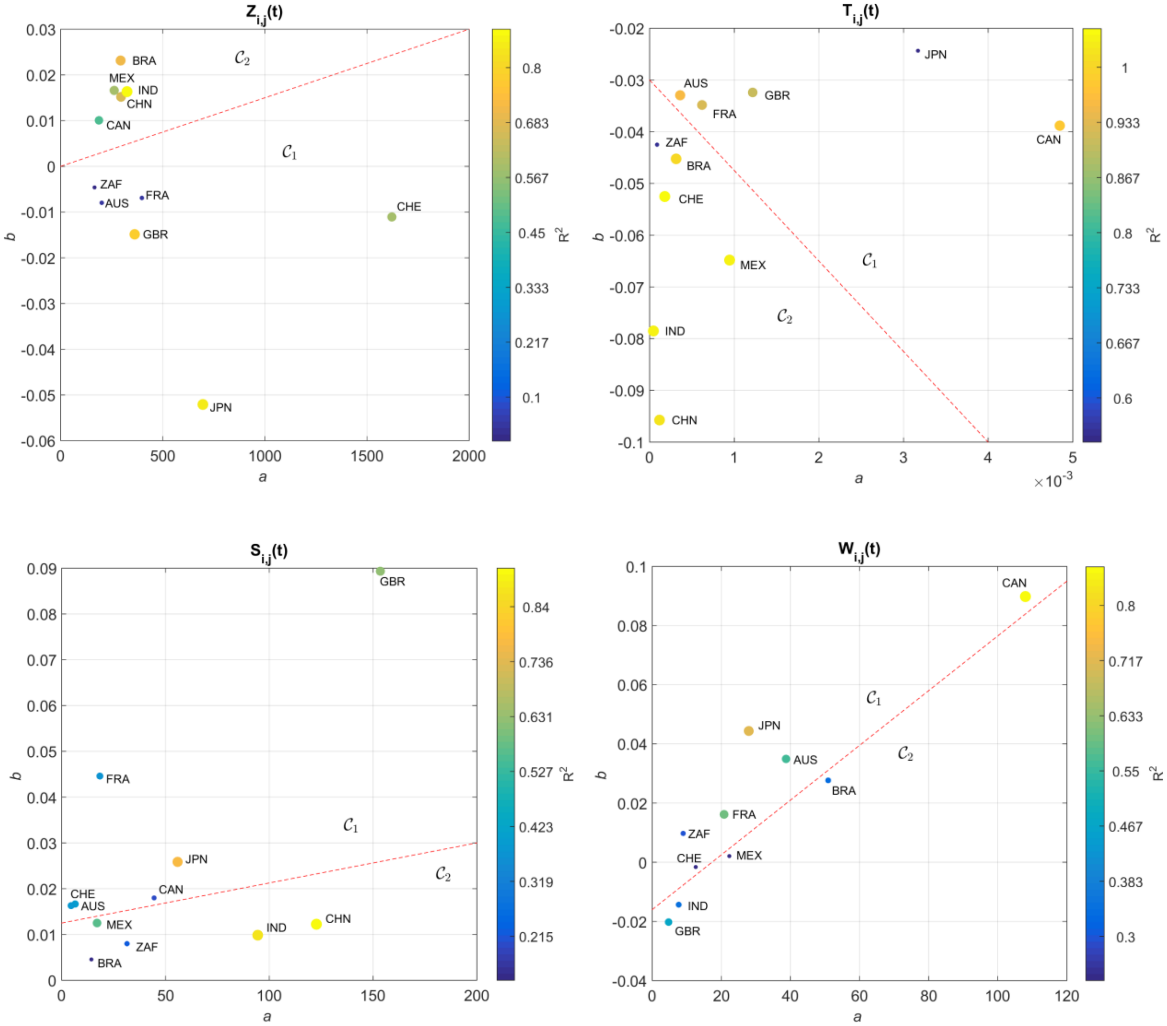
Locus of the trendline parameters (*a*, *b*) in the Cartesian plane, Q, for the determinants *T*_*i*,*j*_(*t*), *S*_*i*,*j*_(*t*), *Z*_*i*,*j*_(*t*) and *W*_*i*,*j*_(*t*), and the 11 countries analyzed, *t* ∈ [1960, 2015]. The diameter and color of the circles are proportional to the value of *R*^2^. The dashed line delimits 2 clusters.

The locus (*a*, *b*) obtained for *T*_*i*,*j*_(*t*), *S*_*i*,*j*_(*t*), *Z*_*i*,*j*_(*t*) and *W*_*i*,*j*_(*t*) are represented in Fig 7, on the Cartesian plane, Q. The diameter and color of the circles are proportional to the value of *R*^2^. For *F*_*i*,*j*_(*t*) no reliable data could be found.

We verify that, for *S*_*i*,*j*_(*t*), the same clusters, $C_{1}$ and $C_{2}$, already identified by the HC analysis of the SS trajectories, emerge. However, for *T*_*i*,*j*_(*t*), *Z*_*i*,*j*_(*t*) and *W*_*i*,*j*_(*t*) the clustering is not complete. For example, with *T*_*i*,*j*_(*t*) we observe that Switzerland moved from $C_{1}$ towards $C_{2}$. Regarding *Z*_*i*,*j*_(*t*) we verify that Canada and South Africa swap location. For *W*_*i*,*j*_(*t*) we observe that United Kingdom and South Africa changed cluster, while China is not represented due to insufficient available data.

These determinants are relevant and shed light into important aspects. Nevertheless, we can ask if it is possible to take advantage of advanced computational techniques and develop new visualization perspectives and indices. This approach will be discussed in the next sub-section.

#### 4.2.2 The distance-based approach

We consider below the variables GDP *per capita*, *x*_1,*i*_(*t*), annual exports of goods and services, *x*_2,*i*_(*t*), and life expectancy at birth, *x*_3,*i*_(*t*). As before, for reference the USA time series are adopted.

For comparison, the distance between country time series *i* and *j* at year *t* is calculated by two distinct measures, namely the arc-cosine and the Canberra distances [63], defined as:

$$d_{ij}^{A}\left(t\right)=\text{arccos}\left(\frac{\sum_{k=1}^{3}x_{k,i}\left(t\right)x_{k,j}\left(t\right)}{\sqrt{\sum_{k=1}^{3}x_{k,i}^{2}\left(t\right)\sum_{k=1}^{3}x_{k,j}^{2}\left(t\right)}}\right),$$ (5)

$$d_{ij}^{C}\left(t\right)=\overset{3}{\underset{k=1}{\sum }}\alpha_{k}\frac{\left|x_{k,i}\left(t\right)-x_{k,j}\left(t\right)\right|}{\left|x_{k,i}\left(t\right)\right|+\left|x_{k,j}\left(t\right)\right|}.$$ (6)

The parameters $\alpha_{k}\in R^{+}$ correspond to weighting factors in the Canberra distance. They are not considered in the arc-cosine distance (5) since it is not influenced by them. Moreover, the USA is adopted for reference, yielding *i* = 1, ⋯, 11, and *j* = 12 (i.e. the USA). The period of analysis is *t* ∈ [1960, 2015] years.

The first index $d_{ij}^{A}$ measures the angle between two vectors. It is the trigonometric inverse function of the cosine correlation that denotes an angular metric based on the normalized inner product. The second index $d_{ij}^{C}$ describes the distance between the two objects in terms of relative values. Therefore, both expressions are insensitive to the absolute amplitude of each of the indices.

**Fig 8:**
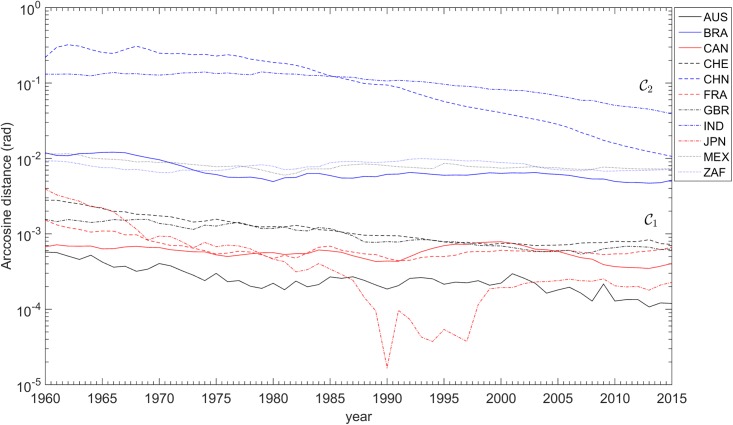
The arc-cosine distances $d_{ij}^{A}$ toward the USA versus time, for each of the 11 countries analyzed, *t* ∈ [1960, 2015]. The clusters $C_{1}$ = {AUS, CAN, CHE, FRA, GBR, JPN, USA} and $C_{2}$ = {BRA, CHN, IND, MEX, ZAF} are visible.

**Fig 9:**
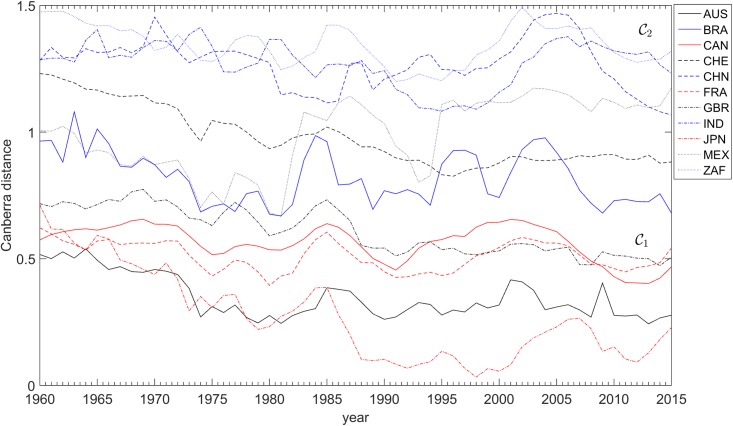
The Canberra distances $d_{ij}^{C}$ toward the USA versus time, for each of the 11 countries analyzed, *t* ∈ [1960, 2015], and weights *α*_*k*_ = {1, 1, 1}. The clusters $C_{1}$ = {AUS, CAN, CHE, FRA, GBR, JPN, USA} and $C_{2}$ = {BRA, CHN, IND, MEX, ZAF} are visible.

**Fig 10:**
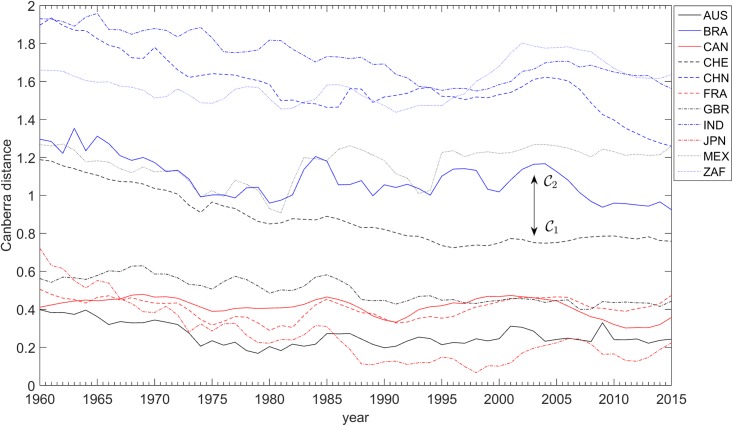
The Canberra distances $d_{ij}^{C}$ toward the USA versus time, for each of the 11 countries analyzed, *t* ∈ [1960, 2015], and weights $\alpha_{k}=\{\frac{7}{3},\frac{1}{3},\frac{1}{3}\}$. The clusters $C_{1}$ = {AUS, CAN, CHE, FRA, GBR, JPN, USA} and $C_{2}$ = {BRA, CHN, IND, MEX, ZAF} are visible.

The variations of the distances $d_{ij}^{A}\left(t\right)$ and $d_{ij}^{C}\left(t\right)$ over the years are depicted in Figs 8, 9 and 10, respectively. For the $d_{ij}^{C}\left(t\right)$ we use two distinct sets of weights, namely *α*_*k*_ = {1, 1, 1} and $\alpha_{k}=\{\frac{7}{3},\frac{1}{3},\frac{1}{3}\}$. As mentioned above, the distance $d_{ij}^{A}\left(t\right)$ is insensitive to changes in weights, contrary to $d_{ij}^{C}\left(t\right)$, where giving more weight to the GDP *per capita* has the effect of enlarging the gap between countries in clusters $C_{1}$ and $C_{2}$.

**Fig 11:**
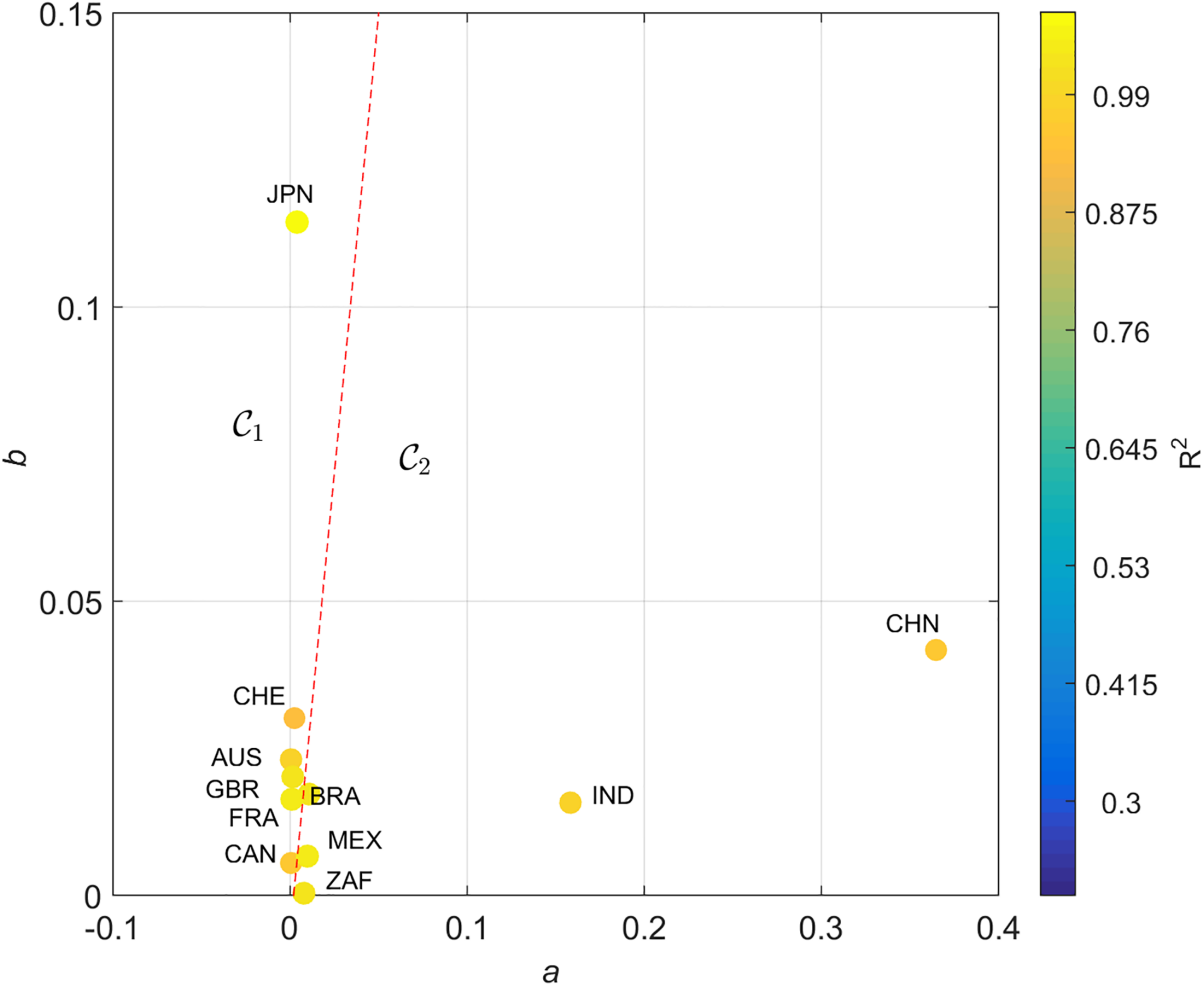
Locus of the trendline parameters (*a*, *b*) in the Cartesian plane, Q, for the arc-cosine distance $d_{ij}^{A}$ and the 11 countries analyzed, *t* ∈ [1960, 2015]. The diameter and color of the circles are proportional to the value of *R*^2^. The dashed line delimits the clusters $C_{1}$ and $C_{2}$.

**Fig 12:**
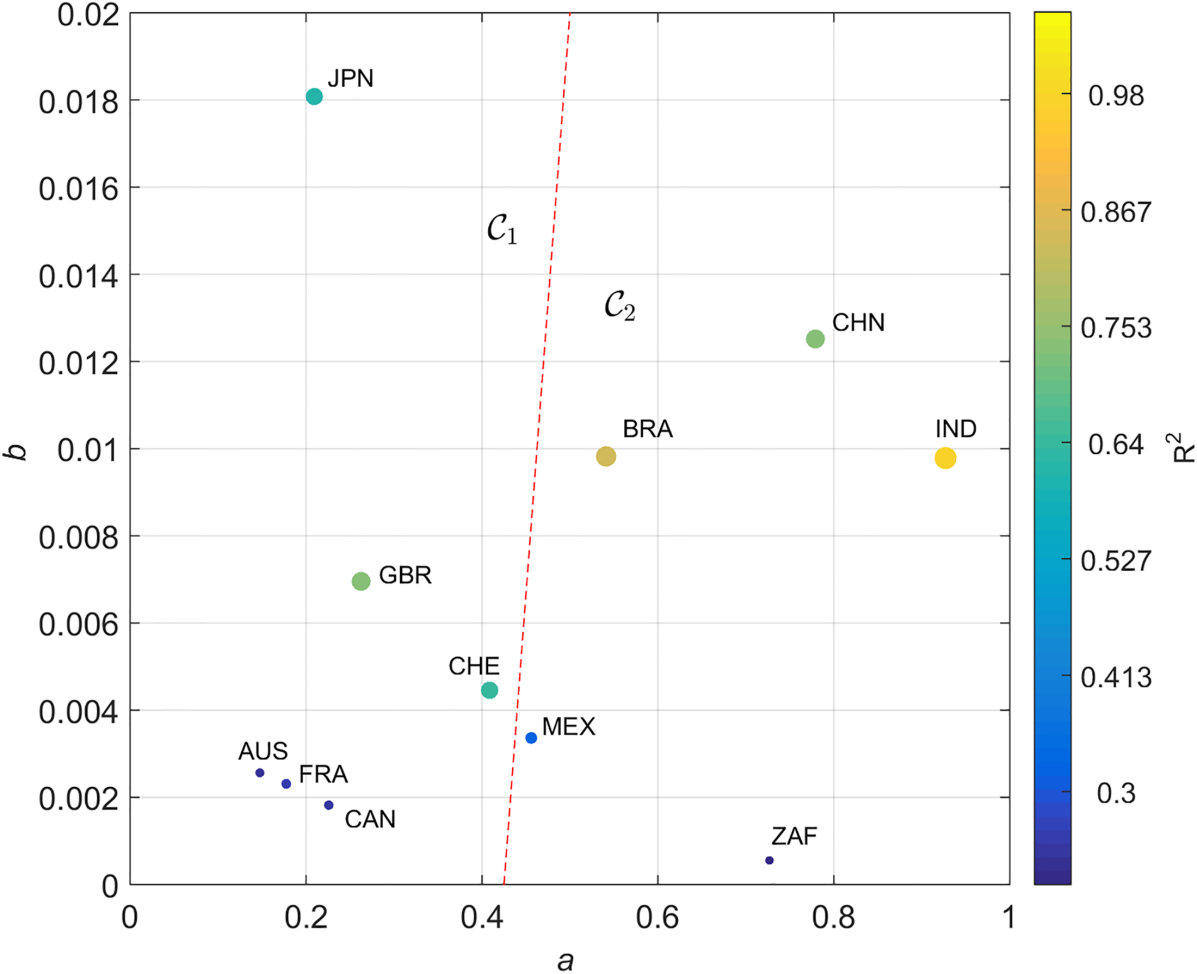
Locus of the trendline parameters (*a*, *b*) on the Cartesian plane, Q, for the Canberra distances $d_{ij}^{C}$ and the 11 countries analyzed, *t* ∈ [1960, 2015] and equal weights. The diameter and color of the circles are proportional to the value of *R*^2^. The dashed line delimits the clusters $C_{1}$ and $C_{2}$.

A good fit for all cases is verified, with the coefficient of determination varying in the interval *R*^2^ ∈ [0.48, 0.97] for $d_{ij}^{A}\left(t\right)$ and *R*^2^ ∈ [0.30, 0.82] for $d_{ij}^{C}\left(t\right)$. The locus (*a*, *b*) for the two distances is represented in Figs 11 and 12, respectively, on the Cartesian plane, Q. The diameter and color of the circles are proportional to the value of *R*^2^.

Distances $d_{ij}^{A}\left(t\right)$ and $d_{ij}^{C}\left(t\right)$ capture distinct dynamic characteristics of the global economic evolution. In both cases, the cluster trees of Fig 4 have a close correspondence with the (*a*, *b*) locus. Moreover, the general layout of the parameters suggests their representation in a distinct coordinate system. In this line of thought, we represent (*a*, *b*) in the hyperbolic plane [64]. Given a point {(*x*, *y*): *x* > 0, *y* > 0} in the first quadrant of the Cartesian plane, Q, its representation in the hyperbolic plane, H, {(u,v):u∈R,v>0}, is calculated through the Q→H transform given by:

$$u=\text{ln}\left(\sqrt{\frac{x}{y}}\right),$$ (7)

$$v=\sqrt{xy},$$ (8)

where the parameter *u* represents the hyperbolic angle to (*x*, *y*) and *v* denotes the geometric mean of *x* and *y*.

**Fig 13:**
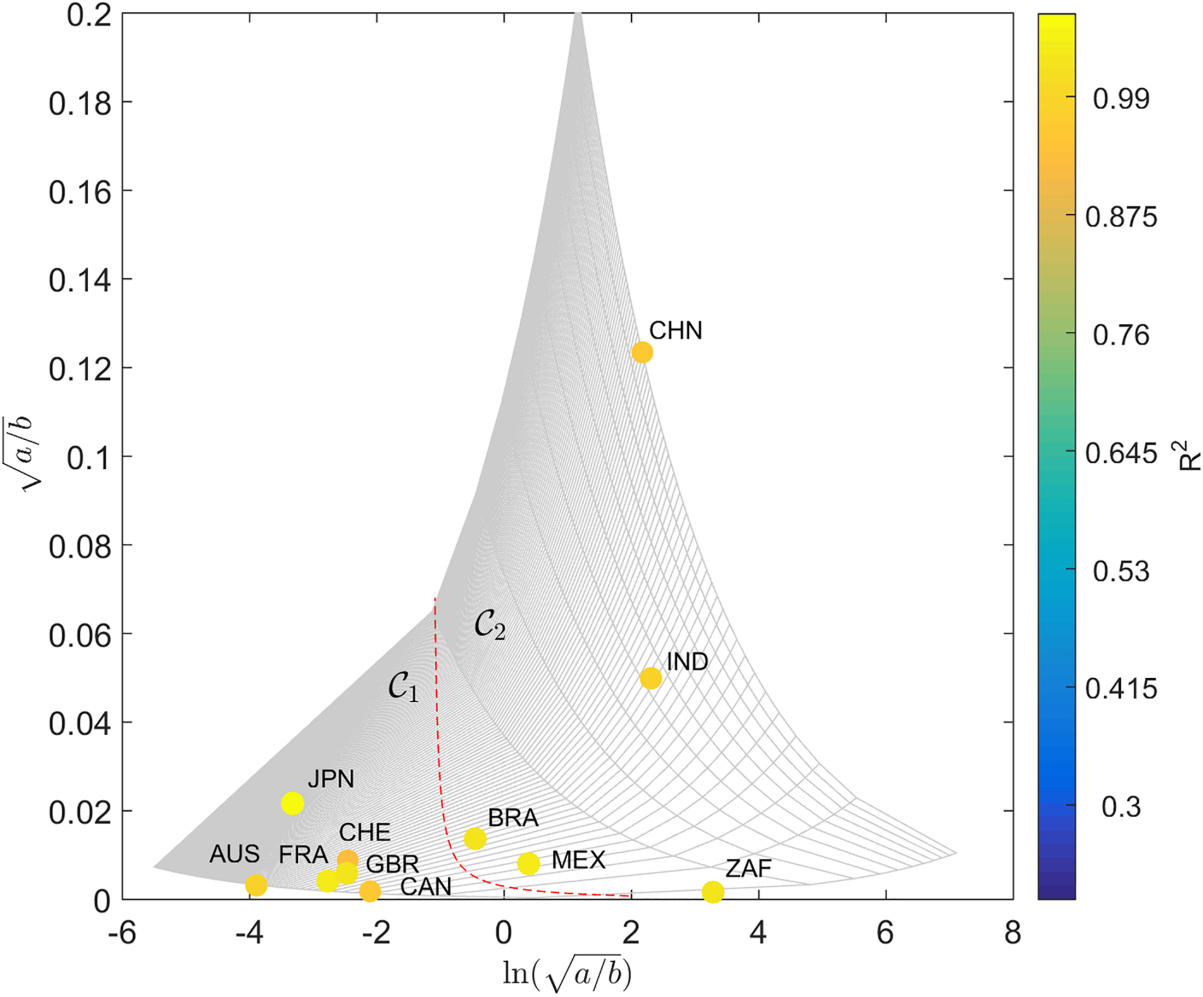
Locus of the trendline parameters (*a*, *b*) in the hyperbolic plane H for the arc-cosine distance $d_{ij}^{A}$ and the 11 countries analyzed, *t* ∈ [1960, 2015]. The diameter and color of the circles are proportional to the value of *R*^2^. The dashed line delimits the clusters $C_{1}$ and $C_{2}$.

**Fig 14:**
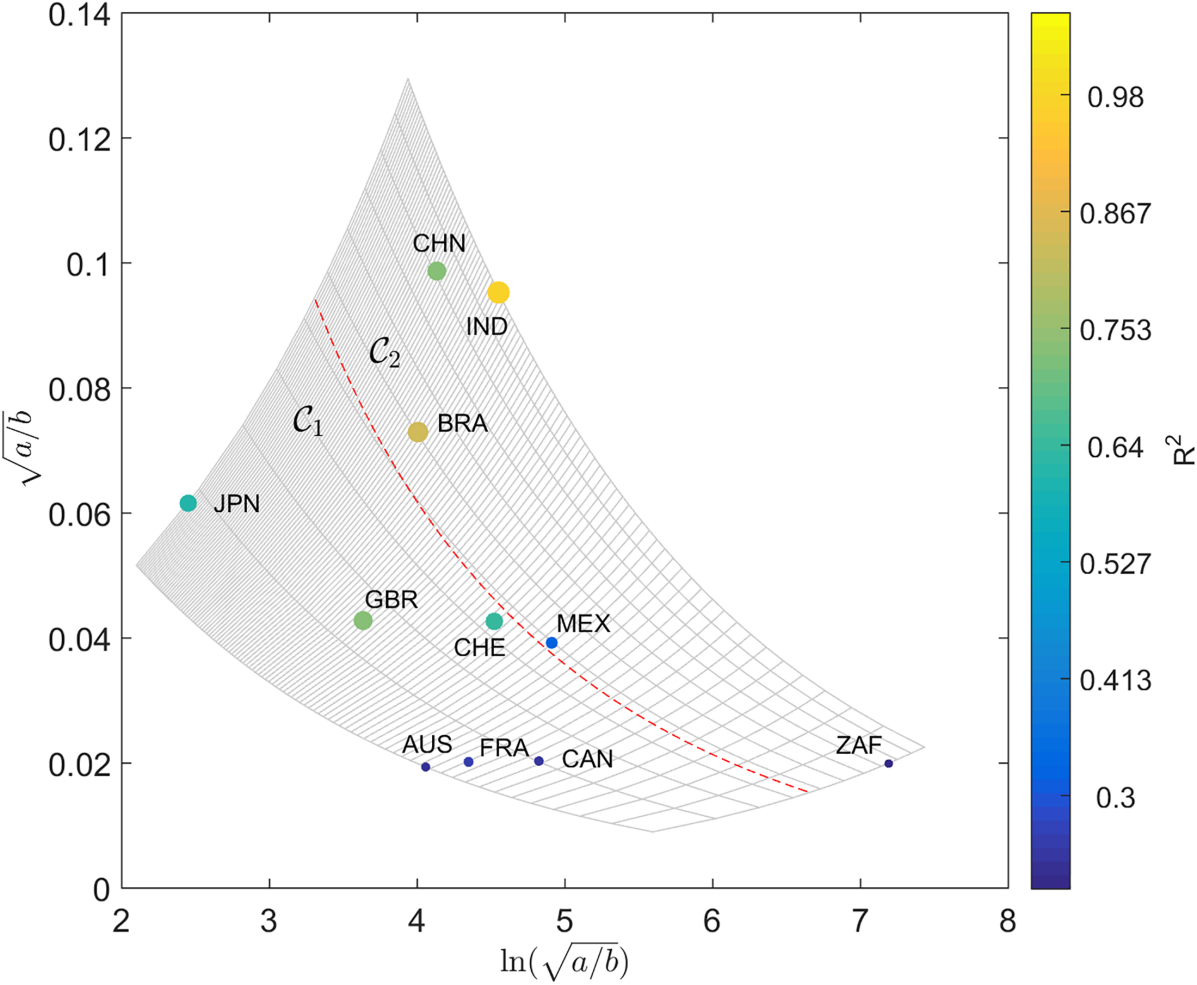
Locus of the trendline parameters (*a*, *b*) in the hyperbolic plane H for the Canberra distance $d_{ij}^{C}$ and the 11 countries analyzed, *t* ∈ [1960, 2015] and equal weights. The diameter and color of the circles are proportional to the value of *R*^2^. The dashed line delimits the clusters $C_{1}$ and $C_{2}$.

Figs 13 and 14 describe the locus (*a*, *b*) for $d_{ij}^{A}$ and $d_{ij}^{C}$ in H. In Fig 13 the mapping (7) and (8) produces a simple trapezoid (gray lines) of the Cartesian grid, and the discrimination between clusters may follow the standard geometric logic. However, in Fig 14 the nonlinear transformation yields a thin and long curved strip. Therefore, in this case we need to be careful and follow the gridlines more closely. In spite of this additional difficulty, we verify that the hyperbolic representation produces a superior representation, in the perspective of understanding the positioning of the two clusters $C_{1}$ and $C_{2}$.

## 5 Discussion

Under a three-variable characterization, some national economies had similar performances, making it difficult to disentangle their individual paths. This means that business-cycle behavior similarities existed among those partners (European and American countries, including the USA). This result confirms the conclusions on 19 countries, by Negro and Otrok [65] “the average correlation of the entire set of countries has not changed at all in the past 35 years”. India, China, and South Africa seem dissimilar when compared with the European and American partners, which seem to be much more unified in business-cycle behavior. Switzerland’s different identity in the set may evoke its peculiar character of not belonging to the European Union and the Euro monetary currency, and its high degree of economic and financial openness thanks to the large weight of exports of goods and services, namely tourism and banking. The most volatile behavior of population variation has occurred in Switzerland and South Africa, two countries of considerable immigration evolution.

Results obtained show that the highest GDP *per capita* performance belongs to Switzerland, Australia, and the North American countries (Canada and USA). The champion of openness is Switzerland. Figures also reveal how difficult it is to disentangle each national profile from the USA case for long. For this reason, Fig 8 records all estimations, presented as $d_{ij}^{A}$, the arc-cosine distance biases from the USA.

In Fig 8, the closer a country is to the horizontal axis, the more synchronized its business-cycles are to those of the USA. The chart is quite clear in demonstrating the great similarity (or co-movement) of most of the countries, and also the regular synchronization with the USA. Three clubs of synchronization may be distinguished.

A first one, the less synchronized with USA, includes China and India, the two great Asian empires and civilizations. However, both have been in a trend of synchronization. The Chinese trend after 1990 in the hyper-globalization period has led the country to levels of distance that are similar to those of the second club of countries.

This second club of countries, comprising the South American countries of Mexico and Brazil, and South Africa, strongly synchronized with the USA during the Globalization G2. Having experienced large waves of business-cycle fluctuations that prevented their economies from a clear proximity to the USA in the 1980s and 1990s, they have proceeded to distance levels that are similar to those of the Chinese economy but lower than those of the third club.

European partners, Canada, Japan, and Australia always formed the club with greatest co-movement with the USA. They synchronized during G2, and their economies are now closely integrated with the rhythm of the USA economic fluctuations [66]. Among them, Japan has been the most volatile, but also the closest in periods such as the early 1990s, the early 2000s, and the new millennium.

These conclusions are historically robust. The American bubble before the 2007 crisis had no parallel in the Chinese economy, as Fig 9 shows. However, synchronization with America began soon after 2007, demonstrating the global character of the current problems. Japan is now synchronized with the USA’s business cycle, too.

For an overall view, Fig 11 describes all partners’ synchronization velocities, using arc-cosine distances. Relative positions are a synthesis for the entire period.

All comments are confirmed in looking at the Canberra distances $d_{ij}^{C}$ between each of the 11 countries analyzed and the USA. Fig 9 describes all countries and the Canberra bias between the national economies here considered and the USA case. The closer they are to the horizontal axis, the more synchronized they are with the USA. Diverging from the USA means de-synchronizing, thereby expressing the countries’ own cyclical behavior.

During G2 it is quite clear that there was a co-movement: confirming Diebold and Rudebusch [67], Fig 12 clearly shows partners’ synchronization velocities, using the Canberra distance. The European Union co-integration effects are here reflected, as Camacho et al. [51] conclude by means of using multidimensional scaling mapping. Business-cycle volatility characterized all the European economies throughout the entire 1980s and 1990s period, following the fall of the Berlin wall. This means that all partners had a business cycle of their own. The ravages of the Soviet Union’s breakup brought the collective hope of reversing some of the political consequences of WW2 on the continent. The desire to unify the divided German nation, and the political aim of extending European hegemony over the Eastern countries that had been under the Soviet influence, required considerable hyper-globalization. Independent business-cycle features are major drivers of collective national achievement, with the impact of international contagion effects. Great turbulence and chaotic business-cycle volatility was the result. The new-millennium’s highly-correlated business cycles of the European monetary union, which were born from a new dream after the end of communism, reveal the extent to which a boost in national economies can bring interactions to influence (or to be influenced by) prosperity in other partners’ business cycles [68]. Contagion effects from the American sub-prime crisis are also evident. Nowadays, multinationals’ concerns with a global recession are reflected in the World Trade Organization fears, and “more recently in public resistance to trade and investment agreements such as the Transatlantic Trade and Investment Partnership, and the Trans-Pacific Partnership” [46].

## 6 Conclusions

This paper proposed a computational approach to the modeling of world economies based on time series. The proximity of standards of living and business cycles to those of the USA were approached using a number of variables for 12 large-economy countries. Several description methods were considered, namely SS representation, HC techniques, and distance based-measures followed by trendline approximation. The behavior of the USA was assumed as the pattern for comparison.

The multivariate techniques for computational analysis showed that there is a long-run co-movement of these 11 large economies with the USA’s performance. The cross-state deviations from the USA’s level of activity measured by means of arc-cosine and the Canberra distances reflect historical moments: the Latin America crisis of the 1980s, the European countries’ volatility during Europe’s extension to the East, and the Japanese issues of the 1990s are main regional cyclical problems visible in the results of the modeling procedure.

In the new millennium co-movement with the American pattern has increased. China shows the most dissimilar business-cycle behavior, but is also synchronizing with the American business cycles, especially over the last decade and a half.

Synchronization may be the result of global power, or perhaps, as some might suggest, mutual influence. Is it instead the case that the USA’s business cycles are converging toward China’s fluctuations? In a word, no. The USA’s 2008 subprime mortgage screen belongs to its pattern in which all other trading partners see their fortunes reflected, including China and the Eurozone countries with their current unemployment crisis.

The data available until 2015 may foretell an imminent slower economic growth path for all partners, in a de-globalization process.

The mathematical and computational exercises reported in this work also help to disentangle euphoria about globalization by revealing how financially interconnected the world has become in the new millennium, and also how difficult it is to say that the 2008-2015 convergence and synchronization is the turning point for de-globlization. The computational methods reported here are untried, it must also be said, but they yield results that are of interest, align well with the historical record, and bring evidence to the issue of how far USA imbalances can be seen as calling the cadence to which integrated markets march—progressing from independent strides of individuals to ever greater lock-step of a unit.
